# Rifaximin Reduces Markers of Inflammation and Bacterial 16S rRNA in Zambian Adults with Hepatosplenic Schistosomiasis: A Randomized Control Trial

**DOI:** 10.4269/ajtmh.17-0637

**Published:** 2018-02-12

**Authors:** Edford Sinkala, Kanekwa Zyambo, Ellen Besa, Patrick Kaonga, Bright Nsokolo, Violet Kayamba, Michael Vinikoor, Rabison Zulu, Martin Bwalya, Graham R. Foster, Paul Kelly

**Affiliations:** 1 Department of Internal Medicine, University Teaching Hospital, Lusaka, Zambia;; 2 Department of Internal Medicine, Tropical Gastroenterology & Nutritional Group, University of Zambia, Lusaka, Zambia;; 3 Department of Medicine, University of Alabama at Birmingham, Birmingham, Alabama;; 4 Centre for Infectious Disease Research in Zambia, Lusaka, Zambia;; 5 Paediatric Centre of Excellence Laboratory, University Teaching Hospital, Lusaka, Zambia;; 6 Blizard Institute, Barts & The London School of Medicine, Queen Mary University of London, London, United Kingdom

## Abstract

Cirrhosis is the dominant cause of portal hypertension globally but may be overshadowed by hepatosplenic schistosomiasis (HSS) in the tropics. In Zambia, schistosomiasis seroprevalence can reach 88% in endemic areas. Bacterial translocation (BT) drives portal hypertension in cirrhosis contributing to mortality but remains unexplored in HSS. Rifaximin, a non-absorbable antibiotic may reduce BT. We aimed to explore the influence of rifaximin on BT, inflammation, and fibrosis in HSS. In this phase II open-label trial (ISRCTN67590499), 186 patients with HSS in Zambia were evaluated and 85 were randomized to standard care with or without rifaximin for 42 days. Changes in markers of inflammation, BT, and fibrosis were the primary outcomes. BT was measured using plasma 16S rRNA, lipopolysaccharide-binding protein, and lipopolysaccharide, whereas hyaluronan was used to measure fibrosis. Tumor necrosis factor receptor 1 (TNFR1) and soluble cluster of differentiation 14 (sCD14) assessed inflammation. 16S rRNA reduced from baseline (median 146 copies/µL, interquartile range [IQR] 9, 537) to day 42 in the rifaximin group (median 63 copies/µL, IQR 12, 196), *P* < 0.01. The rise in sCD14 was lower (*P* < 0.01) in the rifaximin group (median rise 122 ng/mL, IQR-184, 783) than in the non-rifaximin group (median rise 832 ng/mL, IQR 530, 967). TNFR1 decreased (*P* < 0.01) in the rifaximin group (median -39 ng/mL IQR-306, 563) but increased in the non-rifaximin group (median 166 ng/mL, IQR 3, 337). Other markers remained unaffected. Rifaximin led to a reduction of inflammatory markers and bacterial 16S rRNA which may implicate BT in the inflammation in HSS.

## INTRODUCTION

The most significant cause of mortality and morbidity in patients with hepatosplenic schistosomiasis (HSS) is variceal bleeding as a result of portal hypertension.^1–3^ The commonest cause of portal hypertension globally is cirrhosis but in the tropics, HSS dominates.^4,5^ In Zambia, the prevalence of schistosomiasis can reach 77% in endemic parts of the country,^6^ whereas in the western part of the country, Mutengo et al. found active infection of 42% of adults screened and Payne et al. found seroprevalence to be 88% in the same district.^7,8^

Bacterial translocation (BT) in cirrhosis contributes to portal hypertension and is the main cause of mortality.^9^ BT is defined as the movement of bacteria from the intestine to the mesenteric lymph nodes and/or portal vein then into the systemic circulation.^10–14^ In cirrhosis, endotoxins and other vasoactive mediators, because of BT, cause a vasodilatory effect and increases splanchnic blood flow which in turn leads to increased portal hypertension^15^ and may precipitate variceal hemorrhage. Others have speculated that endotoxins result in release of endothelin-1 after cytokine activation. Endothelin-1 then leads to increased portal pressure by increasing vascular resistance.^16,17^ Use of antibiotics in managing spontaneous bacterial peritonitis (SBP) in cirrhosis is widely advocated.^13–15^ We know that SBP occurs because of BT in cirrhosis^18^ but it is unclear if SBP occurs in HSS.

BT remains unexplored in HSS and we are not sure whether it contributes to portal hypertension in these patients. A short report from Western Kenya suggested that there is increased endotoxaemia in patients exposed to *Schistosoma mansoni*,^19^ but it was not established if patients had portal hypertension. Another study suggested that BT may play a role in postoperative infectious complications in patients with schistosomal portal hypertension.^20^ In a case–control study, we recently demonstrated that HSS is characterized by inflammatory cytokines that may be as a result of BT from the gut.^2^ Proof of causation is difficult to secure, so we attempted to confirm the occurrence of BT in HSS by giving rifaximin orally to reduce gut microbial contents. Rifaximin is a broad-spectrum antibiotic and is active against gram-negative and gram-positive bacteria including anaerobes and is minimally absorbable.^18,21^ Therefore, effects of downstream pathophysiology would implicate BT. We postulated that if the markers of BT during treatment with rifaximin are reduced, this would be strong evidence that bacteria are translocating from the gut and driving inflammation.

We therefore set out to investigate if giving rifaximin to patients with HSS would reduce the markers of BT and consequently reduce the systemic inflammatory markers. We also explored the effect of rifaximin on hyaluronan (HA), a fibrotic marker in these patients.

## MATERIALS AND METHODS

### Study design.

This was a phase II open-label randomized control trial, but all primary endpoints were measured in the laboratory by staff members who were unaware of the treatment allocation. Consent from the patients was obtained in writing and verbally. Randomization was carried out using a list of codes prepared by an independent advisor with a random number generator. Sequential patients were enrolled and their treatment allocation was decided by referring to the code list.

### Study population.

Patients were recruited in the Department of Internal Medicine and Endoscopy Unit of the University Teaching Hospital, Lusaka, Zambia, between January 2014 and August 2016. Inclusion criteria were hematemesis and/or splenomegaly, varices, 18 years and above, and positive serology for schistosomiasis. Exclusion criteria were cirrhosis, seropositive for human immunodeficiency virus, hepatitis B virus, hepatitis C virus, or inability to give consent.

### Study medication.

Of the 186 patients who were evaluated, 85 patients fulfilled the criteria and were randomized to rifaximin 600 mg orally twice per day with standard care, or standard care only, and followed up for 42 days. Standard care for both groups comprised praziquantel 40 mg per kg body weight given orally in divided doses over 1 day and propranolol 40 mg three times per day, escalating the dosage upward as tolerated and aiming for reduction in resting pulse rate to below 60 beats/minute. Drugs were self-administered orally at home. Adherence to rifaximin was monitored by self-report.

### Evaluation criteria.

All patients were to complete a 42-day follow-up. The primary outcomes in this study were changes in plasma inflammatory, BT, and fibrotic markers over a 42-day period of rifaximin. The secondary outcomes were response to beta blockers, variceal rebleeding episodes, and mortality. Safety was assessed by adverse events and the laboratory tests.

### Assessments.

A questionnaire was administered at baseline and at the end of the follow-up period. The questionnaire captured demographic data including history of contact with water bodies. Medical history in the questionnaire included hematemesis, abdominal pain, abdominal swelling, yellowing of eyes, fever, and number of tablets of rifaximin missed during the clinical trial. Physical examination was done at baseline and at day 42 and this information was recorded in the questionnaire. Examination included all systems with much emphasis on the liver and the gastrointestinal system. The information in the questionnaire was useful in analyzing various outcomes of the clinical trial.

Ten milliliters of blood was drawn from each patient at baseline and day 42 and collected in tubes containing heparin anticoagulant. Full blood count was measured using a Sysmex 800i analyzer, Kobe, Japan. The rest of the blood sample was centrifuged at 3,000 rpm at 4°C for 5 minutes. The plasma was aliquoted into triplicates and stored at −80°C. The inflammatory markers measured were tumor necrosis factor receptor 1 (TNFR1) and soluble cluster of differentiation 14 (sCD14). To measure BT, 16S rRNA, lipopolysaccharide (LPS), and lipopolysaccharide-binding protein (LBP) were used, whereas HA was used to measure fibrosis.^2^ Limulus amoebocyte lysate chromo was used to detect LPS in plasma (Associates of Cape Cod Incorporated, Falmouth, MA). DNA was extracted using plasma Qiagen DNA extraction kit according to the manufacturer’s instructions. Quantitative polymerase chain reaction was used to quantify DNA of the 16S rRNA gene as an indication for BT. LBP was also used as a biomarker of BT,^2^ using enzyme-linked immunosorbent assay (ELISA) (R&D Systems, Abingdon, United Kingdom) at 2-fold dilution. HA was measured by ELISA (R&D Systems) at 80-fold dilution.

TNFR1 was used as a measure of tumor necrosis factor alpha activity in blood^22^ using ELISA (R&D Systems) at 10-fold dilution. sCD14 was also determined by ELISA (R&D Systems) at 400-fold dilution. ELISA assays were run in duplicate with appropriate controls and analyzed on a Biotek EL 800 ELISA plate reader using Gen5 1.10 software.

### Statistical analysis.

Assuming 95% confidence interval, with a reduction in BT in HSS, a sample size of 40 patients in each group was sufficient with power of 80% to detect a significant difference.

Data were entered into Microsoft Excel and imported to STATA version 13.1 (Stata Corp, College Station, TX) and GRAPHPAD PRISM 6.01 (GraphPad Software, San Diego, CA) for analysis. Intention to treat analysis was used. For description purposes, we used median with interquartile range because most of these data were not normally distributed. To compare the blood tests between the groups, we used the Mann–Whitney test for unpaired data. To compare measurements from baseline to day 42 within the group, we used Wilcoxon matched-pairs signed-rank test. A *P* value of less that 0.05 was considered significant.

### Ethical approval.

This study was approved by the University of Zambia Biomedical Research Ethics Committee (ref: 006-07-12) and Zambia Medicine Regulatory Board (clinical trial number: CT046/13). The trial was registered internationally (ISRCTN67590499).

## RESULTS

**Figure 1.**
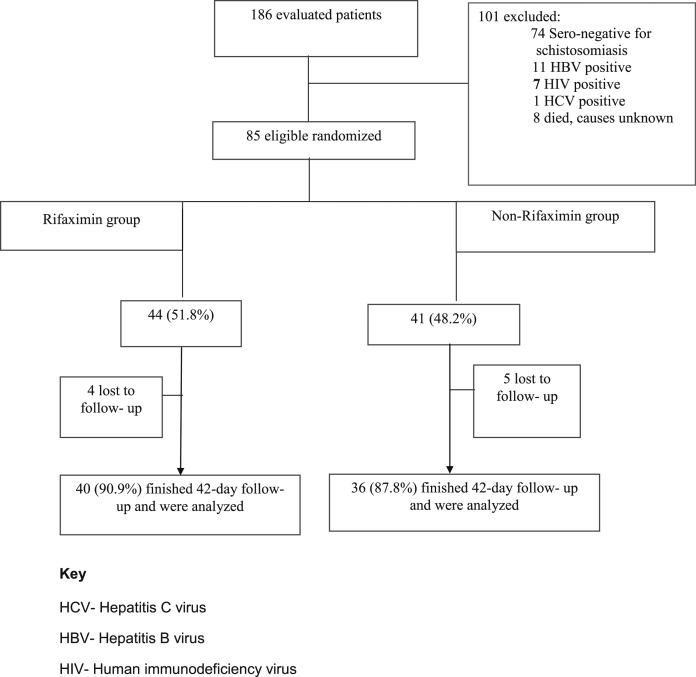
Trial flowchart.

One hundred and eighty-six patients were evaluated and of the 101 ineligible patients, most of them were seronegative for schistosomiasis (Figure 1); although many of these may have had schistosomiasis in the past, we required seropositivity to ensure homogeneity of the trial population. The rifaximin and non-rifaximin groups were similar with respect to demographic and most laboratory data (Table 1). The commonest cause of referral to the hospital was hematemesis (Table 1). At baseline, most of the patients had pancytopenia, but there was an increase in hemoglobin in both groups after 42 days of follow-up; other full blood count indices remained the same (Tables 1 and 2). There were apparent good responses to beta blockade in both rifaximin and non-rifaximin groups after the 42-day follow-up (Table 2). Ten (23%) patients in rifaximin group and seven (24%) patients in the non-rifaximin group had ascites at baseline. Even after 42 days of follow-up, eight patients had ascites on physical examination, five (12%) patients in the rifaximin group, and three (8%) patients in the non-rifaximin group. All patients had normal body temperature at baseline and after 42 days in both groups.

**Table 1:** Baseline demographic and clinical data, inflammatory, translocation, and other blood markers

Variable	Rifaximin group		Non-rifaximin group	
Age (years)	42 (30, 52)		38 (31,43)	
Gender	Females	24	Females	16
	Males	20	Males	25
BMI (kg/m^2^)	21.6 (19.9, 23.2)		21.5 (20.3, 23.8)	
No. of cases with history of hematemesis	Yes	27	Yes	26
	No	17	No	15
Pulse rate (beats/minute)	76 (66, 82)		70 (64, 76)	
Hb (g/dL)	7.5 (6.2, 11.3)		9.2 (6.6, 11.0)	
WCC (×10^9^/L)	2.7 (1.6, 3.5)		1.8 (1.6, 3.0)	
PLT (×10^9^/L)	49 (31, 78)		44 (24, 64)	
MCV (fl)	80 (67, 87)		79 (70, 83)	
MPV (mm)	12.7 (11.2, 13.9)		12.1 (10.8, 13.6)	
16S rRNA copies/µL	129 (23, 499)		51 (19, 112)	
LPS (ng/mL)	74 (38, 157)		154 (45, 649)	
LBP (ng/mL)	27 (23, 30)		43 (38, 50)	
TNFR1 (ng/mL)	1,443 (1,232, 1,911)		1,495 (1,238, 1,657)	
sCD14 (ng/mL)	2,402 (1,930, 2,798)		1,487 (1,299, 1,964)	
Hyaluronan (ng/mL)	125 (57, 189)		102 (71, 176)	

BMI = body mass index; Hb = hemoglobin; LBP = lipopolysaccharide-binding protein; LPS = lipopolysaccharide; MCV = mean cell volume; MPV = main portal vein diameter; PLT = platelets; sCD14 = soluble cluster of differentiation 14; WCC = white cell count; TNFR1 = tumor necrosis factor receptor 1.

All parameters are represented as median with interquartile range in parenthesis.

**Table 2:** Assessment of pulse and full blood count at baseline and day 42

Variable	Rifaximin group baseline	Rifaximin group day 42	*P* value	Non-rifaximin group baseline	Non-rifaximin group day 42	*P*
Pulse rate (beats/minute)	76 (66, 82)	65 (60, 72)	< 0.01	70 (64, 76)	66 (62, 70)	0.07
Hb (g/dL)	7.5 (6.2, 11.3)	11.5 (9.0,13.0)	0.01	9.2 (6.6, 11.0)	10.3 (8.5, 12.4)	0.03
MCV (fl)	80 (67, 87)	76 (66, 86)	0.09	79.0 (70.2, 82.7	79.4 (68.1, 91.9)	0.82
WCC (×10^9^/L)	2.7 (1.6, 3.5)	2.0 (1.6, 2.5)	0.04	1.8 (1.6, 3.0)	2.2 (1.6, 3.5)	0.67
RBC (×10^12^/L)	3.4 (2.7, 4.3)	4.5 (4.0, 4.9)	0.01	3.6 (3.0, 4.4)	4.3 (3.6, 5.2)	0.06
PLT (×10^9^/L)	49 (31, 78)	42 (28, 72)	0.61	44 (24, 64)	38 (25, 52)	0.74

Hb = hemoglobin; MCV = mean cell volume; PLT = platelets; RBC = red blood cells; WCC = white cell count.

All parameters are represented as median with interquartile range in parenthesis.

### Primary efficacy endpoints.

**Figure 2.**
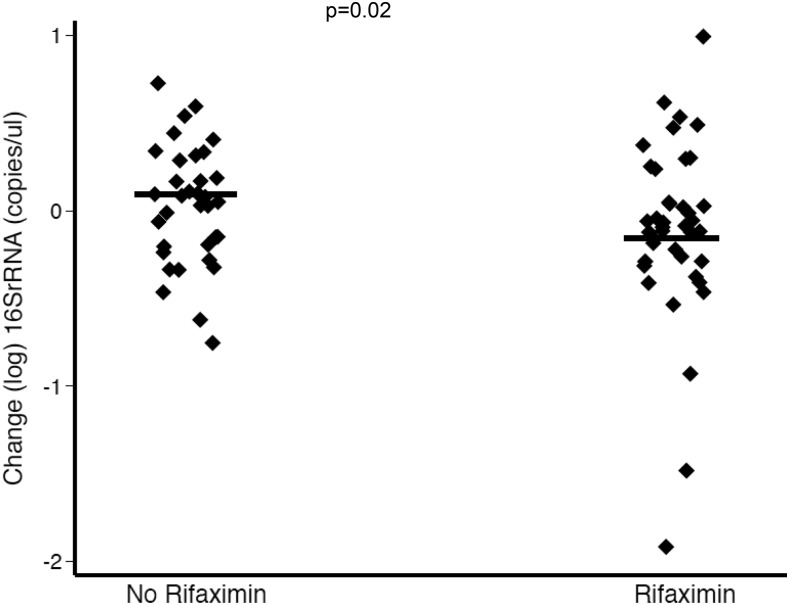
Rifaximin led to the reduction of 16S rRNA copies/µL over 42 days.

**Figure 3.**
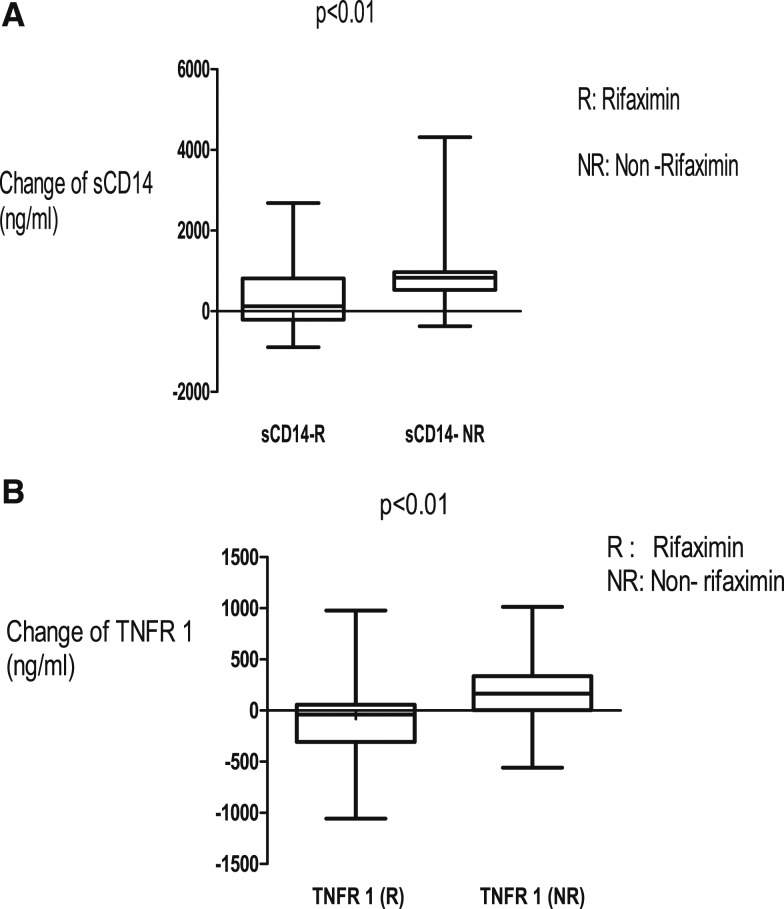
(**A** and **B**) Soluble CD14 (sCD14) and tumor necrosis factor receptor 1 (TNFR1) plasma concentrations fell in the rifaximin group over 42 days.

**Figure 4.**
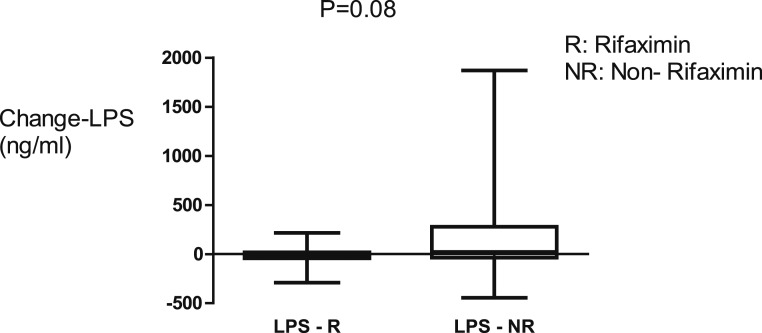
There was no significant change in lipopolysaccharide (LPS) concentrations in either group over 42 days.

**Figure 5.**
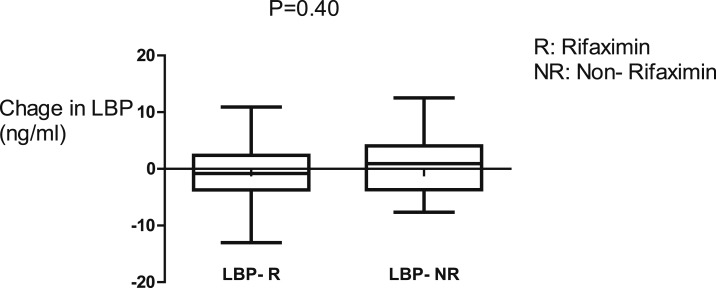
There was no significant change in lipopolysaccharide-binding protein (LBP) concentrations after 42 days in either rifaximin or non-rifaximin groups.

**Figure 6.**
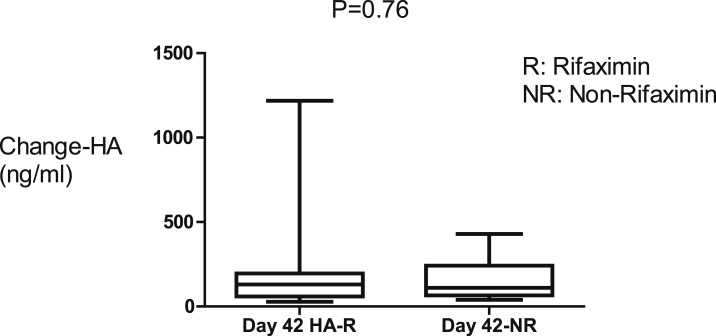
Hyaluronan (HA) concentrations did not change in both rifaximin and non-rifaximin groups.

There was reduction of 16S rRNA, a marker of BT, sCD14, and TNFR1, inflammatory markers for 42 days of rifaximin as shown in Figures 2 and 3. There were no significant changes in the plasma concentrations of LPS, LBP, and HA after 42 days of rifaximin treatment (Figures 4–6).

### Secondary endpoints.

There was good clinical effect of beta blockade (reduction in resting pulse pressure). No patient reported any episode of hematemesis.

### Safety.

There were no major adverse events reported during a 42-day follow-up. One patient in the rifaximin group reported an episode of rectal bleeding that required no intervention. Another patient in the rifaximin group complained of abdominal discomfort a week after starting taking rifaximin, but this resolved without any intervention.

## DISCUSSION

The reduction in inflammatory markers and 16S rRNA copies after 42 days of intestinal decontamination with rifaximin provide evidence that BT may have a clinical association with inflammatory processes in HSS-related portal hypertension. We know that BT is associated with cirrhosis, and this drives the disease process by increasing portal hypertension.^10,11^ Our results suggest that similar mechanisms may also be driving portal hypertension in HSS.

The pronounced reduction of systemic inflammatory markers and the significant reduction of 16S rRNA copies might be explained by the contribution of BT as rifaximin is a nonabsorbable oral antibiotic.^18,23–27^ The minimal reduction of LPS is not inconsistent with this interpretation as LPS is a constituent of gram-negative bacteria only,^28^ and it might be expected that rifaximin would have a greater effect on gram-positive bacterial loads. Among the inflammatory markers that were reduced after 42 days of rifaximin treatment, sCD14 is the marker which other researchers have associated with BT.^24,29–32^ LPS elicits secretion of sCD14 from macrophages including Kupffer cells after binding of LPS to toll-like receptor 4 in the presence of LBP and sCD14.^24,25^ For this reason, activation of any of these markers could reflect BT. TNFR1, another inflammatory marker, also fell after 42 days of rifaximin treatment which is consistent with BT as a driver of systemic inflammation. Tarrats et al.^33^ have associated TNFR1 with liver fibrosis in animal models. LBP did not change much after 42 days of rifaximin in this clinical trial, which is somewhat at odds with the effect we saw in the case–control study.^2^ Other researchers have shown that LBP may also be elevated in gram-positive sepsis in the elderly and those who are obese.^34–36^ In this clinical trial, no patient had obesity and there was no extremity of age. The lack of effect of rifaximin on LBP may have a similar explanation to that for the lack of effect on LPS.

The only fibrotic marker measured in patients in the clinical trial was HA which was not affected by rifaximin. We think that the duration of rifaximin was too short to have any impact on HA, and by the time these patients present to hospital with variceal bleeding fibrosis is well established and likely to be irreversible. HA is an important molecule which is associated with hepatic fibrosis in HSS.^2,37^ In an animal model of cirrhosis, fibronectin, another fibrotic marker, reduced after intestinal decontamination with rifaximin.^38^ It is not clear whether this could translate to humans but our data suggest not in the short term.

The pancytopenia in our patients is consistent with hypersplenism. There was profound thrombocytopenia in both groups but patients did not present with bleeding during follow-up although Arthur and others have described platelet counts of 100 × 10^9^/L or less as being associated with upper digestive bleeding even in asymptomatic HSS patients.^39^

There were no major adverse events associated with rifaximin during the 42-day follow-up period which is in agreement with what others have said about the safety of rifaximin.^18^ The reason for the low variceal rebleeding rate during the 42-day follow-up could be due to adherence to propranolol as confirmed by good beta blockade at the end of 42-day period in both groups compared with baseline. This probably reflects the importance of beta blockers in preventing variceal bleeding in schistosoma-related portal hypertension.^40^

It is a limitation of this study that it was an open-label clinical trial, which is prone to bias, although the laboratory personnel were blinded. Confirmation of compliance to rifaximin in these patients was not possible as rifaximin blood levels could not be measured. The effect of BT on clinical endpoints may also have been underestimated in this clinical trial because the patients were stable and seen as outpatients. They did not have acute variceal bleeds on recruitment and would be predicted to have lower rebleeding rates on follow-up.

## CONCLUSION

We showed that rifaximin led to the reduction of systemic inflammatory cytokines and bacterial 16S rRNA, a direct marker of BT. This suggests that BT may be involved in the pathophysiology of HSS and warrants the further study to demonstrate if rifaximin gives clinical benefit. It would be of interest to study patients recruited immediately after acute variceal bleeds, with clinical endpoints, perhaps as a multicenter placebo-controlled trial on a larger scale.
